# Oncolytic Rhabdovirus Vaccine Boosts Chimeric Anti-DEC205 Priming for Effective Cancer Immunotherapy

**DOI:** 10.1016/j.omto.2020.10.007

**Published:** 2020-10-14

**Authors:** Fanny Tzelepis, Harsimrat Kaur Birdi, Anna Jirovec, Silvia Boscardin, Christiano Tanese de Souza, Mohsen Hooshyar, Andrew Chen, Keara Sutherland, Robin J. Parks, Joel Werier, Jean-Simon Diallo

**Affiliations:** 1 Centre for Innovative Cancer Research, Ottawa Hospital Research Institute, Ottawa, ON, Canada; 2 Department of Biochemistry, Microbiology and Immunology, University of Ottawa, Ottawa, ON, Canada; 3 Laboratory of Antigen Targeting to Dendritic Cells, Department of Parasitology, University of São Paulo, São Paulo, Brazil; 4 Institute for Investigation in Immunology (iii)-INCT, São Paulo, Brazil; 5 Department of Surgery, The Ottawa Hospital, Ottawa, ON, Canada; 6 Regenerative Medicine Program, Ottawa Hospital Research Institute, Ottawa, ON, Canada

**Keywords:** anti-DEC205, rhabdovirus, oncolytic vaccine, Ad5, prime-boost, immunotherapy

## Abstract

Prime-boost vaccination employing heterologous viral vectors encoding an antigen is an effective strategy to maximize the antigen-specific immune response. Replication-deficient adenovirus serotype 5 (Ad5) is currently being evaluated clinically in North America as a prime in conjunction with oncolytic rhabdovirus Maraba virus (MG1) as a boost. The use of an oncolytic rhabdovirus encoding a tumor antigen elicits a robust anti-cancer immune response and extends survival in murine models of cancer. Given the prevalence of pre-existing immunity to Ad5 globally, we explored the potential use of DEC205-targeted antibodies as an alternative agent to prime antigen-specific responses ahead of boosting with an oncolytic rhabdovirus expressing the same antigen. We found that a prime-boost vaccination strategy, consisting of an anti-DEC205 antibody fused to the model antigen ovalbumin (OVA) as a prime and oncolytic rhabdovirus-OVA as a boost, led to the formation of a robust antigen-specific immune response and improved survival in a B16-OVA tumor model. Overall, our study shows that anti-DEC205 antibodies fused to cancer antigens are effective to prime oncolytic rhabdovirus-boosted cancer antigen responses and may provide an alternative for patients with pre-existing immunity to Ad5 in humans.

## Graphical Abstract

**Figure:**
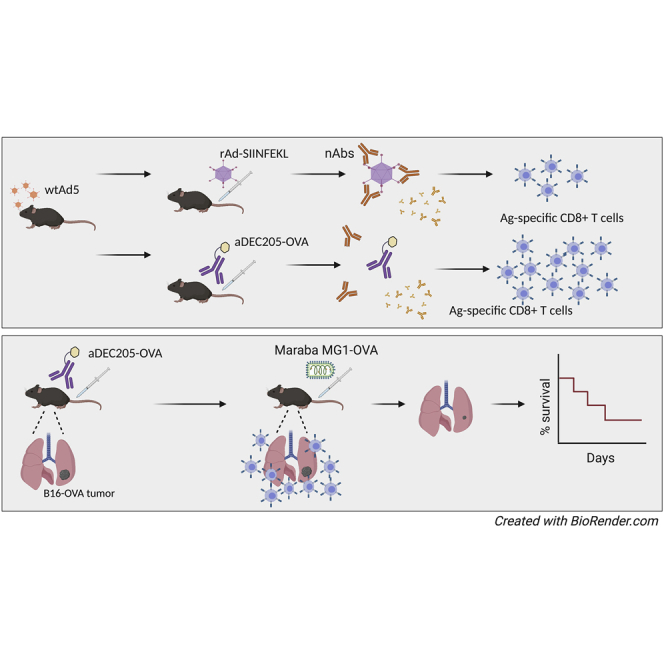

## Teaser

Replication-deficient Ad5 vectors are used for gene therapy and vaccination. Tzelepis and colleagues show that seropositivity to Ad5, highly prevalent in the human population, abrogates an Ad5 vaccination response. Alternatively, they show the efficacy of αDEC205 antibody-antigen fusion in the context of an oncolytic rhabdovirus cancer vaccine targeting mouse melanoma.

## Introduction

As knowledge of the important role played by the immune system in preventing tumor growth in healthy individuals has expanded over the last decades, immunotherapy has emerged as a viable treatment option for cancer.^1^ One form of immunotherapy that has gained recent regulatory approval employs oncolytic viruses (OVs). OVs are live, replicating viruses selected or genetically modified to preferentially target and kill cancer cells while leaving healthy cells relatively unharmed.^2^ This is possible owing to the fact that cancers exhibit many characteristics that are conducive to successful viral replication, such as resistance to apoptosis, increased nucleotide synthesis, and an impaired antiviral response.^3^ OVs elicit their anti-cancer effects through multiple mechanisms and following tumor cell lysis and immunogenic cell death, can trigger anti-cancer immune responses.^4^ In addition to Imlygic, an intratumorally delivered oncolytic herpes simplex virus 1 (HSV-1) strain approved for treatment of late-stage melanoma, many different viruses have been clinically evaluated for their potential as OVs, including many that can be delivered intravenously (i.v.), such as (but not limited to) measles virus,^5^ coxsackie virus,^6^ and rhabdoviruses, like vesicular stomatitis virus (VSV) and the closely related Maraba virus (MG1).^7^ Additional attenuating genetic modifications are generally introduced into OVs in order to increase their safety profile. For example, oncolytic rhabdoviruses are attenuated by deletion of the matrix protein in VSV (termed VSVΔ51) and mutation of components of the matrix and glycoproteins in MG1.^8^ In addition, OVs can be genetically manipulated to encode proteins that either help to establish a productive infection of cancer cells or encode cytokines and/or immunogenic antigens, such as cancer antigens.

It is known that OVs can elicit *in situ* cancer vaccine effects and relieve local immunosuppression through the induction of immunostimulatory cytokines. In this environment, dendritic cells (DCs) can phagocytose dead/dying infected tumor cells and prime an anti-tumor as well as antiviral immune response in the draining lymph node.^9^ However, the heterogeneous nature of cancer has resulted in limited efficacy of OVs as monotherapies and has steered researchers to investigate combinations of these biologics with other therapies that not only enhance OV infection of tumors but also enable anti-tumor immune responses.^10^^,^^11^

Typical vaccination regimens are generally not limited to a single dose and can be made more effective by multiple immunizations. This can involve the administration of additional homologous (matched vaccine) or heterologous (unmatched vaccine) doses.^12^ In the context of cancer vaccines, it has been recently shown that a heterologous prime-boost strategy, where an initial priming dose of an adenovirus virus encoding a cancer antigen is administered, followed by a boosting dose of an oncolytic rhabdovirus encoding the same antigen, can be effective to eradicate tumors.^13^ This strategy has been shown to induce robust and long-term effector T cell responses^14^^,^^15^ and is currently undergoing clinical evaluation for multiple antigens and indications (ClinicalTrials.gov: NCT02285816, NCT02879760, NCT03618953, and NCT03773744).

As a boosting component, oncolytic rhabdoviruses are thought to be uniquely effective because in addition to infecting tumor and breaking local immunosuppression, they efficiently, but nonproductively, infect splenic B cells, which provides an additional source for antigen presentation to DCs, resulting in secondary expansion of T cells.^16^

To prime the oncolytic rhabdovirus boost, current clinical trials employ a nonreplicating adenovirus serotype 5 (Ad5) vector expressing a shared cancer antigen (e.g., MAGE-A3, ClinicalTrials.gov: NCT02285816). Questions regarding the importance of vector seropositivity were raised recently following Merck’s failed phase II clinical trial of a trivalent human immunodeficiency virus (HIV) vaccine delivered in an Ad5 vector.^17^ Indeed, Ad5 seropositivity is sometimes an exclusion criterion in vaccine and gene-therapy clinical trials employing this vector.^18^ Approximately 30%–40% of the North American population is seropositive for Ad5, and this proportion approaches an 85% average globally, posing a potential limitation to the widespread use of Ad5 as a priming vector for the oncolytic rhabdovirus heterologous prime-boost cancer immunotherapy strategy.^19^, ^20^, ^21^

DEC205 is a C-type lectin endocytic receptor highly expressed on certain DC subtypes.^22^ Chimeric antibodies specific to DEC205 fused with an antigen of interest (anti-DEC205 [aDEC205]) have been shown to be an effective strategy to target fused antigens directly to DCs, inducing robust cellular and humoral responses when combined with adjuvants.^23^^,^^24^ To overcome potential issues with Ad5 and other viruses that could be used as priming vectors but that may have the potential to be affected by pre-existing immunity, we hypothesized that chimeric aDEC205 antibodies could provide an effective alternative. In this study, we modeled and evaluated the impact of pre-existing immunity on Ad5-based priming. As proof of concept, we also evaluated a heterologous prime-boost vaccine strategy employing aDEC205-ovalbumin (OVA) as the priming agent, followed by a boost with OVA-expressing oncolytic rhabdoviruses in an experimental model of OVA-expressing B16 melanoma.

## Results

### Pre-existing Immunity to Wild-Type Ad5 (WTAd5) Impairs Generation of a SIINFEKL-Specific Immune Response to Recombinant Ad5-SIINFEKL (rAd5-SIINFEKL)

**Figure 1:**
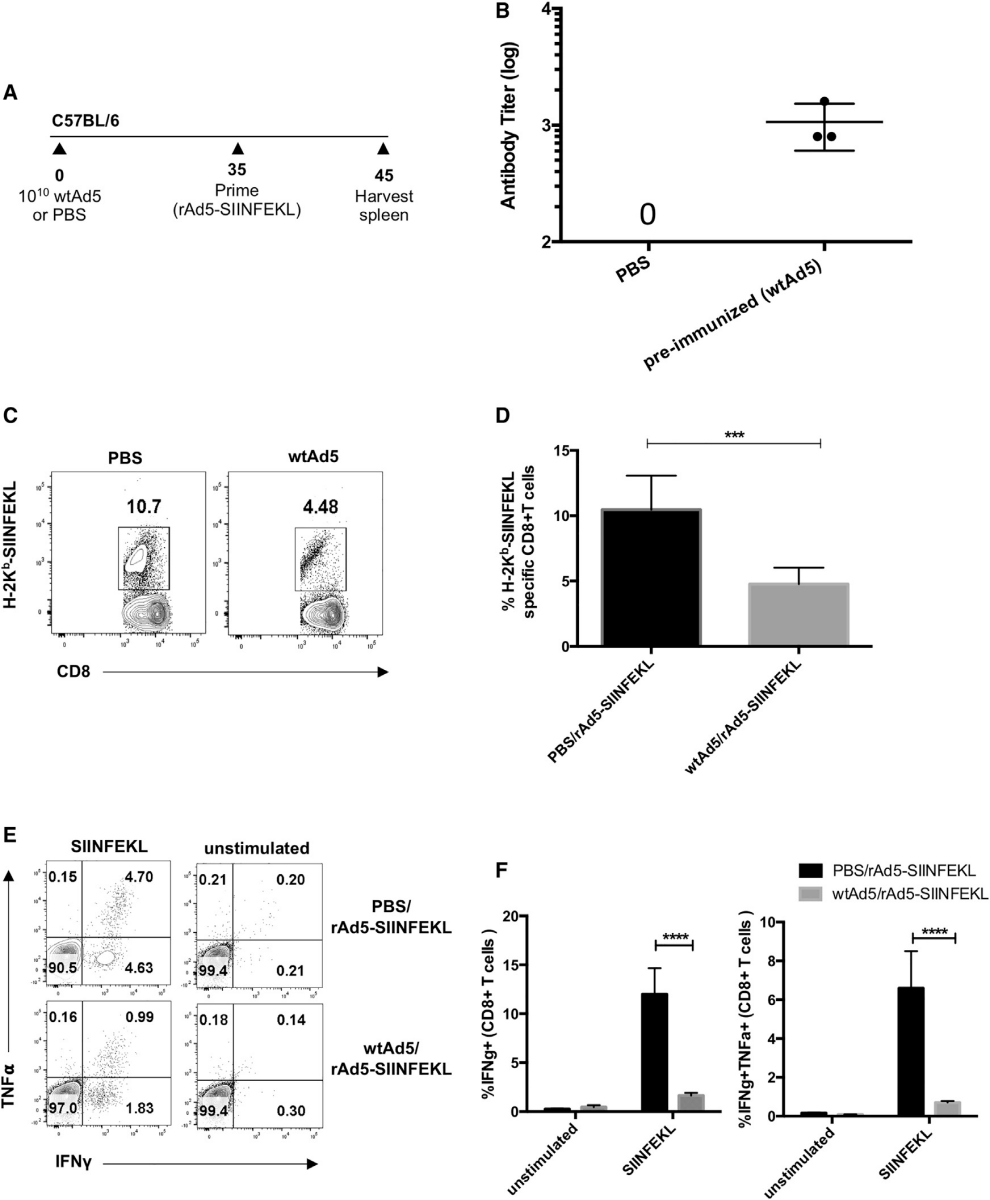
Comparing the SIINFEKL-Specific T Cell Response after i.m. Injection of Priming Agent Recombinant Adenovirus Expressing the SIINFEKL Transgene (rAd5-SIINFEKL) in Mice Modeling Pre-existing Immunity to WTAd5 (A) Naive C57BL/6 mice were injected i.m. on day 0 with 10^10^ PFUs of WTAd5 (n = 7) or PBS (n = 5). After 35 days, mice were injected i.m. with rAd5-SIINFEKL. (B) Anti-adenovirus neutralizing antibody (AdNAbs) titers in mouse sera (n = 3) were determined by neutralization assay, 40 days after administration of WTAd5.10 days after prime, the representative gating (C ) and total percentage of (D) SIINFEKL-specific CD8^+^ T cells in the spleen was determined by H2-K^b^-SIINFEKL pentamer staining.The representative gating (E) and percentage of (F) OVA-specific T cells producing IFN-γ and TNF-α in the spleen was evaluated by flow cytometry. Briefly, splenocytes were stimulated *in vitro* with MHC-I epitope (SIINFEKL) for 5 h, subsequently stained for intracellular production of IFN-γ and TNF-α, and assessed by flow cytometry. ∗∗∗p < 0.001 and ∗∗∗∗p < 0.0001 (two-way ANOVA).

We hypothesized that pre-existing immunity to WTAd5 may negatively affect priming of the immune response induced by rAd5-expressing antigens. To investigate this, we evaluated the capacity of Ad5 encoding the OVA epitope rAd5-SIINFEKL to generate an antigen-specific immune response in mice with pre-existing immunity to WTAd5. To model pre-existing immunity, we immunized naive C57BL/6 mice with 10^10^ plaque-forming units (PFU) of the WTAd5 virus. After 35 days, mice were administered 10^8^ PFUs rAd5-SIINFEKL intramuscularly (i.m.) (Figure 1A). Generation of anti-adenovirus neutralizing antibodies (AdNAbs) in sera of preimmunized mice 40 days postadministration of WTAd5 was confirmed by neutralization assay and was elevated in preimmunized mice (Figure 1B). SIINFEKL-specific CD8^+^ T cell responses were measured 10 days after rAd5-SIINFEKL immunization, the peak time of the adaptive immune response elicited by adenovirus vectors.^25^ We observed a statistically significant decrease from 10% to approximately 5% of splenic SIINFEKL-specific CD8^+^ T cells, depicted by H2K^b^-SIINFEKL pentamer staining, from preimmunized mice compared to control phosphate-buffered saline (PBS) mice (Figures 1C and 1D). To assess CD8^+^ T cell functionality, splenocytes from preimmunized mice and PBS mice were restimulated with SIINFEKL peptide *in vitro* and followed by intracellular cytokine staining (ICS) for interferon (IFN)-γ and tumor necrosis factor (TNF)-α. Again, there was a reduction from an average of 6% to 2% of IFN-γ- and TNF-α-producing CD8^+^ T cells specific to SIINFEKL detected in the splenocytes from preimmunized mice compared to control PBS (Figures 1E and 1F). Together, these results indicate that modeled pre-existing immunity to WTAd5 limits the generation of SIINFEKL-specific cellular responses following rAd5-SIINFEKL immunization in C57BL/6 mice.

### Production and Characterization of aDEC205-OVA

Impaired SIINFEKL-specific immune responses following rAd5-SIINFEKL immunization of C57BL/6 mice modeling pre-existing immunity led us to consider employing an alternative priming agent that would be better able to overcome pre-existing immunity to WTAd5 and other potential alternative viral vectors. Several studies have shown the ability of chimeric aDEC205 antibodies fused to antigens, such as OVA (aDEC205-OVA) or tumor antigens, to elicit strong antigen-specific immune responses in mice when administered with an adjuvant.^26^^,^^27^ To evaluate the use of antigen-fused aDEC205 antibodies as alternative priming agents for heterologous boosting with Oncolytic rhabdovirus (ORV) vectors, we used aDEC205 fused to the model antigen OVA.

To generate the aDEC205 antibodies used in this study, human embryonic kidney (HEK)293T cells were cotransfected with plasmids containing the mouse aDEC205-kappa light chain and the aDEC205 heavy chain fused to the full OVA protein sequence at the carboxyl terminus (or no antigen as a control [aDEC205-empty]). The recombinant antibodies produced following transient transfection in HEK293T cell were purified on protein G Sepharose columns.

**Figure 2:**
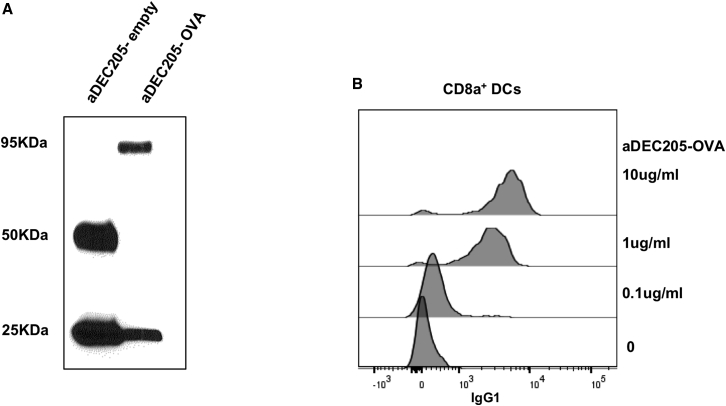
Production and Characterization of aDEC205-OVA (A) aDEC205-OVA and aDEC205-empty were generated by transfection of 293T cells *in vitro* and subsequent purification of the antibody. (A) Final antibody product was reduced by β-mercaptoethanol and verified by immunoblotting for the heavy and light chains. aDEC205-empty shows a heavy chain at 50 kDa and light chain at 25 kDa. aDEC205-OVA shows the heavy chain linked with OVA at 95 kDa, indicating the presence of OVA antigen and a light chain at 25 kDa. (B) A binding assay was performed to verify effective binding of aDEC205-OVA to the DEC205 receptor on CD11c^+^CD8^+^ dendritic cells (DCs) isolated from murine splenocytes. aDEC205-OVA is probed with an anti-IgG1-APC antibody and detected by flow cytometry. The histogram overlay depicts high binding of aDEC205-OVA to CD11c^+^CD8^+^ DCs at concentrations of 10 μg/mL and 1 μg/mL and low binding at 0.1 μg/mL.

The resulting antibodies were characterized by western blot using anti-immunoglobulin G (IgG) antibodies on SDS-PAGE under reducing conditions (Figure 2A). Figure 2A shows that heavy and light chains of the purified recombinant antibodies had the expected size for both the fused antibody (Ab) aDEC205-OVA (∼95 kDa and 25 kDa, respectively) and control antibody aDEC205-empty (50 kDa and 25 kDa, respectively). The capacity of the aDEC205-OVA and aDEC205-empty antibodies to bind to its receptor on the surface of splenic DCs CD11c^+^CD8a^+^ was confirmed with a binding assay.^28^ Incubation of splenocytes from naive C57BL/6 mice with different concentrations of aDEC205-OVA (0.1, 1, or 10 μg/mL) resulted in a dose-dependent binding (Figure 2B) on the surface of splenic CD11c^+^CD8a^+^ DCs (gating strategy shown in Figure S1) expressing the DEC205 receptor. These results indicate that aDEC205-OVA and aDEC205-empty were successfully purified from culture supernatants and that aDEC205-OVA and aDEC205-empty (Figure S2) retain binding capacity to the DEC205 receptor as expected.

### aDEC205-OVA Administered via Intraperitoneal (i.p.) and i.v. Routes Generates Cellular Immune Responses against SIINFEKL

**Figure 3:**
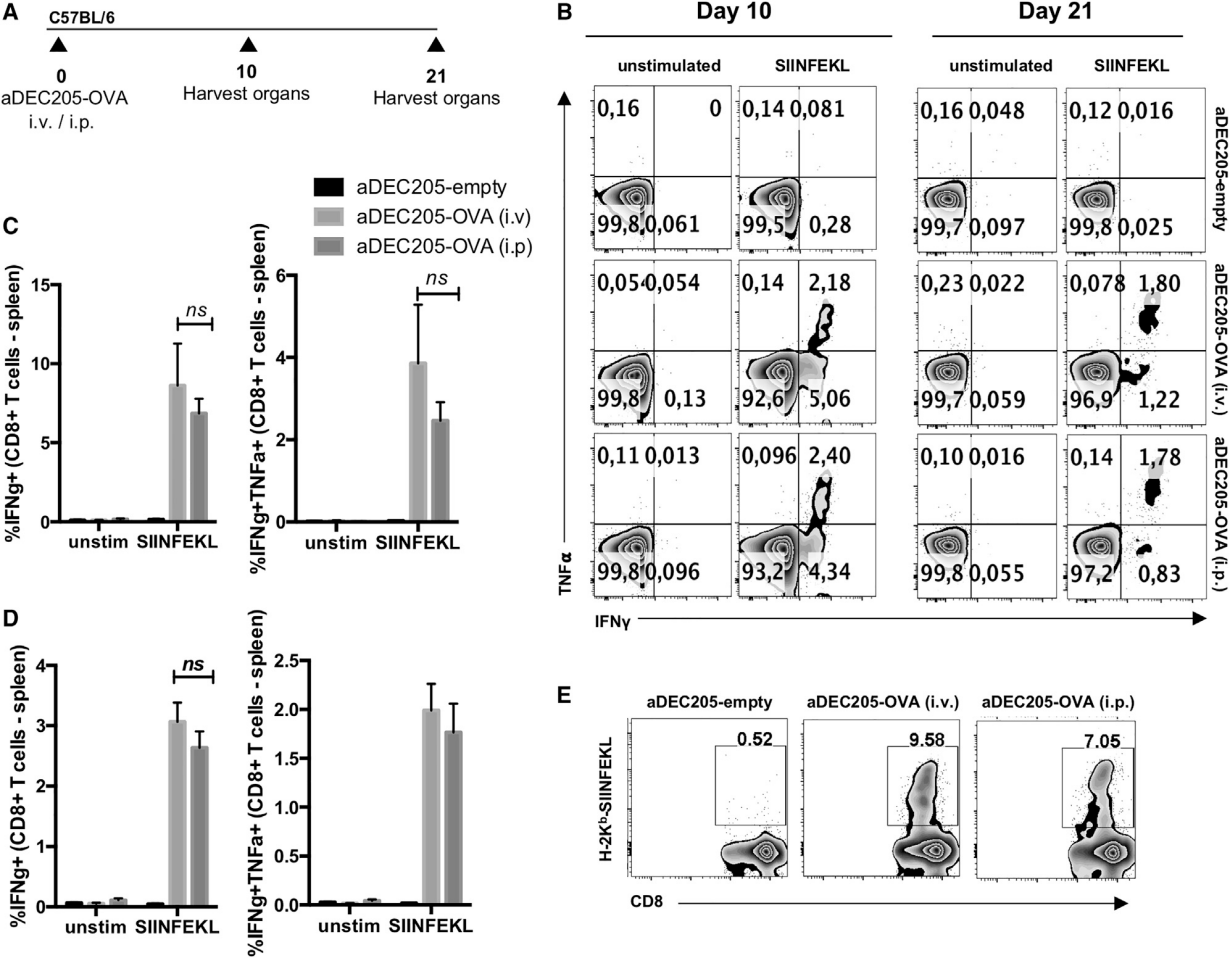
aDEC205-OVA Administered i.v. or i.p. Elicits OVA-Specific T Cells in the Spleen of Immunized Mice (A) Naive C57BL/6 mice were primed with 10 μg of aDEC205-OVA or aDEC205-empty + 50 μg poly(I:C) + 50 μg anti-CD40 i.v. or i.p. The percentage of SIINFEKL-specific T cells producing IFN-γ and TNF-α in the spleen (B) on day 10 (C) and on day 21 (D) was evaluated by flow cytometry. (E) Quantification of SIINFEKL-specific T cells by pentamer staining (H-2K^b^-SIINFEKL) was also assessed in the spleen by flow cytometry at day 21 postinjection. p value was considered nonsignificant (ns) when >0.05 (two-way ANOVA).

Several studies demonstrated the influence of the route of immunization on immune response and disease outcome.^29^ To determine which route of aDEC205-OVA administration leads to the most potent T cell response systemically, we immunized naive C57BL/6 mice i.p. or i.v. with 10 μg aDEC205-OVA or aDEC205-empty, both in combination with 50 μg poly(I:C) and 50 μg anti-CD40. SIINFEKL-specific T cells were evaluated by flow cytometry at 10 and 21 days postimmunization (Figure 3A; gating strategy shown in Figure S3). ICS, after *in vitro* restimulation of lymphocytes with the SIINFEKL peptide, showed that i.v. and i.p. routes of administration elicited statistically similar percentages of IFN-γ- and TNF-α-producing CD8^+^ T cells in the lung and spleen of mice immunized with aDEC205-OVA at days 10 and 21 postimmunization (Figures 3B–3D; Figure S4). Additionally, staining with the H2K^b^-SIINFEKL pentamer showed statistically similar percentages of SIINFEKL-specific CD8^+^ T cells at day 21 in the spleen and lung of mice immunized with aDEC205-OVA when comparing i.v. and i.p. routes of administration (Figures 3E; Figure S4). As expected, no SIINFEKL-specific CD8^+^ T cells were detected in the spleen or lungs of animals immunized with control aDEC205-empty. These results indicate that either route of administration elicits a strong anti-SIINFEKL primary immune response. Ultimately, to model a preferred route of administration in humans, we proceeded to administer aDEC205-OVA i.v. for the remainder of this study.

### aDEC205-OVA Overcomes Barriers Posed by Pre-existing Immunity and Generates Cellular and Humoral Immunity against OVA

**Figure 4:**
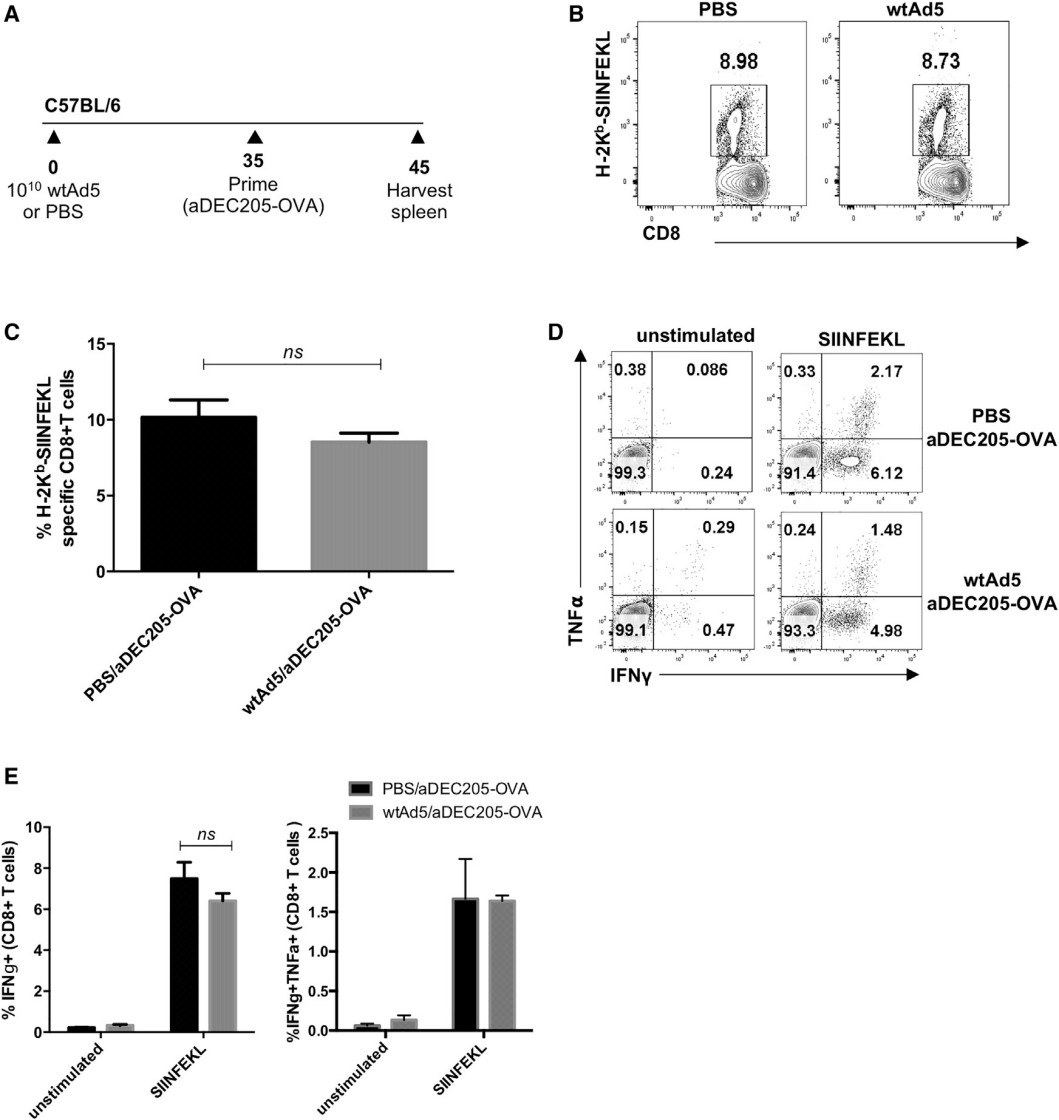
Pre-existing Immunity to Adenovirus Does Not Affect an Immune Response Elicited by aDEC205-OVA Prime (A) Naive C57BL/6 mice were injected i.m. on day 0 with 10^10^ PFUs of WTAd5 or PBS. (B and C) After 35 days, mice were injected i.v. with 10 μg of aDEC205-OVA + 50 μg poly(I:C) + 50 μg anti-CD40. 10 days after priming, the representative gating (B) and percentage of (C) SIINFEKL-specific CD8^+^ T cells in the spleen was determined by H2-K^b^-SIINFEKL pentamer staining. The representative gating (D) and percentage of (E) SIINFEKL-specific T cells producing IFN-γ and TNF-α in the spleen was also evaluated by ICS and flow cytometry. p value was considered nonsignificant when >0.05 (two-way ANOVA).

We next evaluated the ability of aDEC205-OVA to overcome pre-existing immunity to WTAd5 in a C57BL/6 murine model. To model pre-existing immunity, all naive C57BL/6 mice were immunized with WTAd5 35 days prior to the injection of priming agents (Figure 4A). As previously observed, AdNAbs were detected by a neutralization assay in mouse sera 40 days postadministration of WTAd5 and were elevated in preimmunized mice around time of prime (Figure 1B). Pre-existing immunity to WTAd5 did not affect priming with aDEC205-OVA; approximately 9% of SIINFEKL-specific CD8^+^ T cells were observed in the spleen of preimmunized mice and control PBS mice 10 days after prime (Figures 4B and 4C). Furthermore, a similar percentage of splenic IFN-γ- and TNF-α-producing CD8^+^ T cells specific to SIINFEKL was also detected by intracellular staining (Figures 4D and 4E). Together with Figure 1, these results suggest that adjuvanted aDEC205 is an effective prime in the face of pre-existing immunity to WTAd5.

### Heterologous Boosting of aDEC205-OVA Prime with Rhabdovirus-Encoding OVA Potentiates a Cellular and Humoral Immune Response

**Figure 5:**
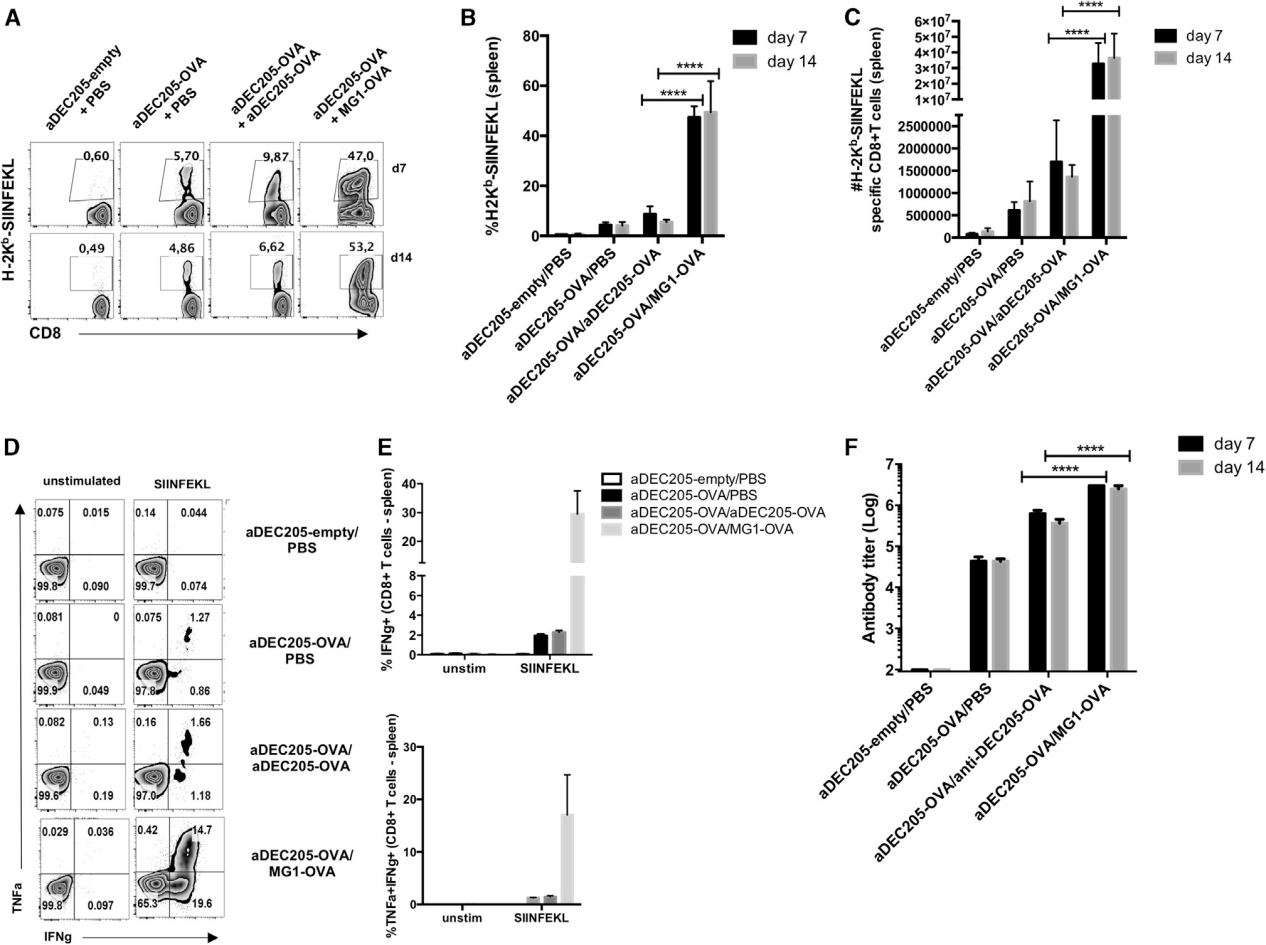
Induction of a Potent Cellular and Humoral OVA-Specific Immune Response after aDEC205-OVA Prime and MG1-OVA Boost C57BL/6 mice were immunized i.v. with 10 μg of aDEC205-OVA or aDEC205-empty + 50 μg poly(I:C) + 50 μg anti-CD40 at day 0. 14 days later, mice were immunized with a boosting dose of PBS, 10 μg aDEC205-OVA i.v. + 50 μg poly(I:C) + 50 μg anti-CD40, or 10^8^ PFUs of MG1-OVA. Spleens were harvested 7 and 14 days following boost to evaluate cellular immune response to prime-boost regimens by flow cytometry (A). The percentage (B) and total number of (C) SIINFEKL-specific CD8^+^ T cells were determined by H2-K^b^-SIINFEKL pentamer staining. At day 14, the representative gating (D) percentage of (E) splenic IFN-γ- and TNF-α-producing CD8^+^ T cells in response to *in vitro* stimulation with 5 μM SIINFEKL peptide was evaluated. (F) The titers of anti-OVA antibodies in the sera of mice were determined by ELISA at day 7 and day 14 after boost. These results are representative of two independent experiments. ∗∗∗∗p < 0.0001 (two-way ANOVA).

Priming with Ad5 encoding a cancer antigen, followed by boosting with ORV vectors, such as MG1 or VSV, expressing the same antigen, induces strong antigen-specific responses, providing survival benefit in various tumor models.^11^, ^12^, ^13^, ^14^, ^15^ Therefore, we tested the ability of the combination aDEC205-OVA prime and MG1-OVA boost in the generation of a SIINFEKL-specific T cell response. To this end, naive C57BL/6 mice were primed (i.v.) with 10 μg aDEC205-OVA or aDEC205-empty, both in combination with 50 μg poly(I:C) and 50 μg anti-CD40 and boosted (i.v.) 14 days later with 10^8^ PFUs of MG1-OVA or 10 μg aDEC205-OVA with 50 μg poly(I:C) and 50 μg anti-CD40 or PBS. 7 and 14 days after boost, lymphocytes were harvested from the spleen and lung and then stained with the H2K^b^-SIINFEKL pentamer. At days 7 and 14 postboost, the greatest expansion of SIINFEKL-specific T cells was observed in the spleen (Figures 5A–5E) and lungs (Figure S5) of animals boosted with MG1-OVA. Similar results were obtained using VSV-OVA (Figures S6A and S6B). Although boost with aDEC205-OVA expanded the antigen-specific cells compared to the group only primed with aDEC205-OVA, the level of expansion was significantly lower compared to MG1-OVA. Humoral immunity was also assessed using mouse sera to quantify OVA-specific IgG by ELISA. The combination of aDEC205-OVA/MG1-OVA prime-boost generated the highest anti-OVA antibody titers compared to other combinations and control groups (Figure 5F). Immunization with aDEC205-OVA/VSV-OVA prime-boost generated similar antibody titers compared to aDEC205-OVA/MG1-OVA prime-boost at day 7 postboost (Figure S6C).

### Heterologous Prime-Boost Vaccine with aDEC205-OVA and Rhabdovirus-Encoding OVA Confers a Survival Advantage in Tumor-Bearing Mice

**Figure 6:**
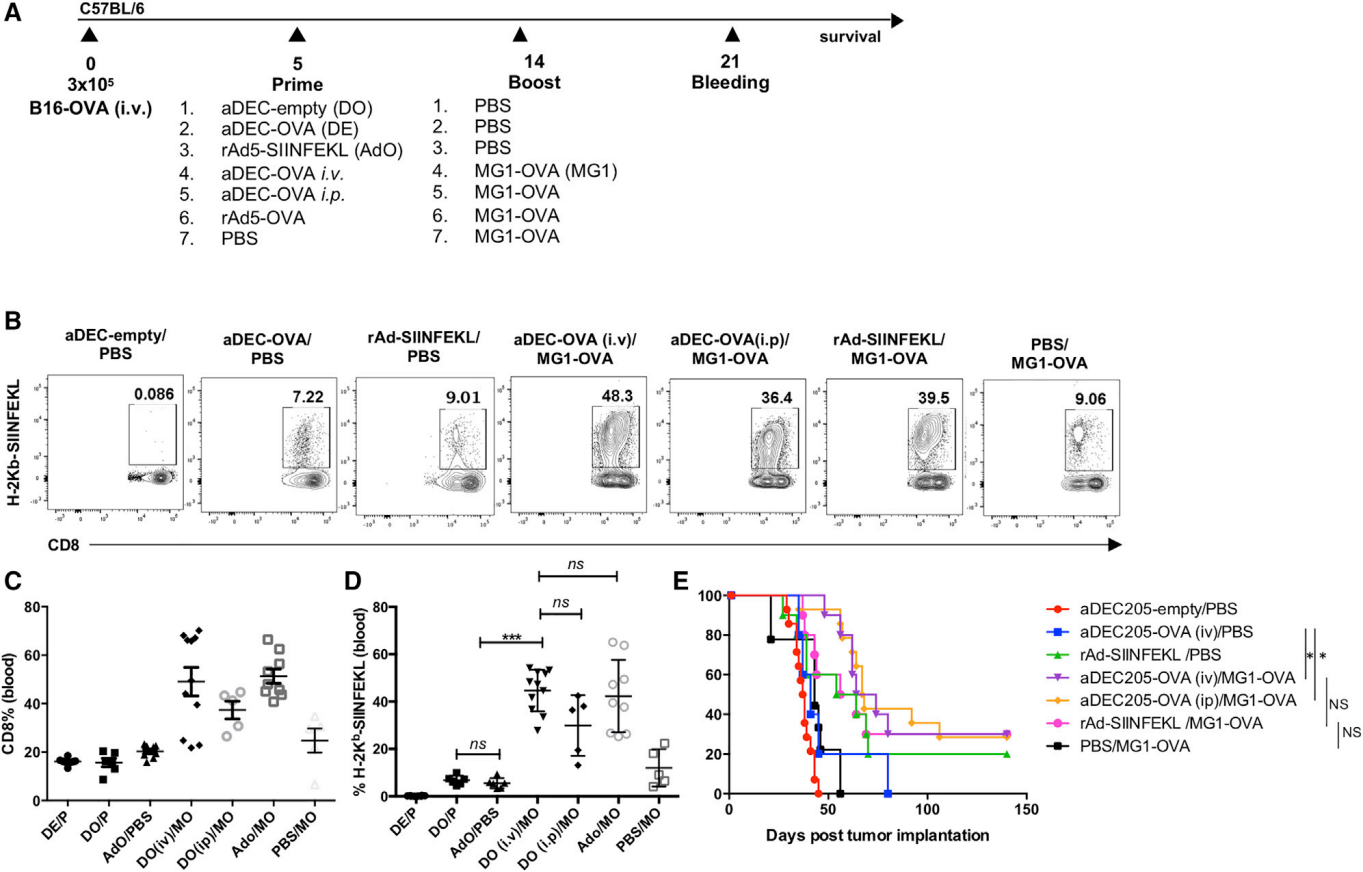
Therapeutic Efficacy of an aDEC205/OVA Prime-Boost Vaccine (A) Schematic representation of immunization schedule. Briefly, C57BL/6 mice received 3 × 10^5^ B16-OVA cells i.v. At day 5, mice were immunized i.v. or i.p. with 10 μg of aDEC205-OVA or aDEC205-empty + 50 μg poly(I:C) + 50 μg anti-CD40, 10^8^ rAd5-SIINFEKL, or PBS. At day 14, mice were immunized with a boosting dose of either PBS or 10^8^ MG1-OVA. (B–D) At day 21 saphenous (saph) bleeds were performed to assess the percentage, by flow cytometry (B), of bulk-circulating CD8^+^ T cells (C) and SIINFEKL-specific CD8^+^ T cells (D), the latter determined by H2-K^b^-SIINFEKL pentamer staining. p value was considered nonsignificant when >0.05; ∗∗∗p < 0.001 (one-way ANOVA). (E) Mice were monitored for survival 140 days post-B16-OVA implantation. Data from three independent survival experiments are pooled. p value was considered nonsignificant when >0.05; ∗p < 0.05 (log-rank Mantel-Cox).

We next evaluated the therapeutic efficacy of the aDEC205-OVA/MG1-OVA prime-boost vaccine in an experimental model of lung metastasis. Briefly, 3 × 10^5^ B16-OVA cells were injected i.v. in C57BL/6 mice, and different primes were administered 5 days post-B16-OVA tumor implantation (Figure 6A). Generation of SIINFEKL-specific T cell responses was evaluated by H2-K^b^-SIINFEKL pentamer staining of blood 7 days after boost. The heterologous prime-boost combination employing aDEC205-OVA or rAd5-OVA as a prime generated the greatest percentage of circulating antigen-specific T cells. Interestingly, whereas different routes of administration of the aDEC205-OVA prime (i.v. versus i.p.) did not significantly impact priming responses (Figure 3), there was a trend for a higher magnitude of a SIINFEKL-specific CD8^+^ T cell response generated after prime with aDEC205-OVA administered i.v. compared to aDEC205-OVA prime administered i.p. (Figures 6B and 6D). In general, all OVA-targeted heterologous prime-boost regimens led to improved survival of tumor-bearing mice, with rAd5-OVA/MG1-OVA and aDEC205-OVA/MG1-OVA regimens being the most effective (30% complete remission). The administering of a prime-boost of aDEC205-OVA/VSV-OVA also resulted in the generation of greater SIINFEKL-specific CD8^+^ T cells and improved survival of tumor-bearing mice (Figures S6B, S6D, and S6E). Cured mice were rechallenged with a subcutaneous injection of 2 × 10^6^ B16-OVA cells (data not shown); no mice previously cured by any prime-boost regimen developed tumors, thus confirming that anti-SIINFEKL responses were long lasting and conferred protection against recurrent tumors.

## Discussion

Cancer immunotherapy has emerged as a promising alternative to conventional cancer treatments. Therapeutic strategies that actively stimulate the immune system to reject tumors have grown to include diverse platforms, including immune-modulating antibodies,^30^ small molecules,^31^^,^^32^ as well as genetically engineered bacteria,^33^ cells,^34^ and viruses.^7^ OVs are attracting increasing interest as multi-mechanistic platforms for immunotherapy, owing, in part, to the recent approval of Imlygic for the treatment of melanoma and to the possibility of combining OVs with antibodies targeting immune checkpoints.^35^ Indeed, it is increasingly recognized that OVs have significant potential as part of combination therapy regimens.^36^

In this study, we have further explored one such combination strategy consisting of a heterologous prime-boost, where the priming and boosting vectors share a similar tumor antigen and where the boosting vector is an oncolytic rhabdovirus.^37^ This is a strategy that is now under phase I/II clinical evaluation using a nonreplicating Ad5 as a priming vector and oncolytic MG1 as a boosting vector. In contrast with repeat dosing with the same vector (homologous vaccination), this heterologous prime-boost approach has been shown to skew the immune response from antiviral to anti-tumor, promoting long-lasting anti-tumor immunity.^12^^,^^38^ The secondary immunization with rhabdovirus, which preferentially infects tumors, not only induces oncolysis but also boosts the primary anti-tumor adaptive immune response and breaks immune tolerance.^8^

Although nonreplicating Ad5 is an effective and well-validated vector for vaccination, pre-existing immunity to Ad5, resulting from prior exposure to WT adenoviruses in humans, can potentially limit its effectiveness in clinical trials.^16^^,^^17^ Indeed, we found that administration of rAd5-SIINFEKL in preimmunized mice led to both significantly lower percentages of SIINFEKL-specific CD8^+^ T cells (Figures 1C and 1D) and a reduction in their functionality (Figures 1E and 1F).

As an alternative to Ad5, we demonstrate here, in line with other studies, that i.p. or i.v. administration of aDEC205-OVA generates antigen-specific and functionally robust anti-SIINFEKL T cells, as well as humoral immunity toward OVA.^22^^,^^25^^,^^39^ However, as a standalone vaccination agent, we observed that aDEC205-OVA did not perform as well as rAd5-SIINFEKL in terms of controlling B16-OVA tumors (Figure 6E) and generating activated (IFN-γ^+^, TNF-α^+^), SIINFEKL-specific CD8^+^ T cells, even though the numbers of SIINFEKL-specific T cells were similar with both primes (Figure 6B). This difference could relate to dosing inequivalence between aDEC205 relative to Ad5, however something that is difficult to establish, owing to differences in how immune responses are initiated with the two vaccination methods. However, the dose of aDE205-OVA used in this study is within the range of the human equivalent dose of what is being evaluated in clinical trials employing aDEC205 (ClinicalTrials.gov: NCT01834248 and NCT01127464).^40^^,^^41^ In comparison, Ad5 was administered at a higher human equivalent dose than what is administered in current clinical trials, further illustrating the potential of aDEC205 over Ad5. Additionally, pre-existing immunity to WTAd5, as evidenced by the presence of AdNAbs (Figure 1B), strongly decreased the ability of rAd5-SIINFEKL to produce an immune response against the SIINFEKL antigen but predictably bore no impact on the ability of aDEC205-OVA to generate functional anti-SIINFEKL CD8^+^ T cells.

Consistent with other studies, we found that heterologous boosting with an oncolytic rhabdovirus, such as MG1-OVA, amplifies antigen-specific immunity in the spleen at days 7 and 14 to a higher extent than homologous boosting, for example, with aDEC205-OVA (Figure 5; Figure S5).^15^^,^^36^ All heterologous regimens tested conferred a survival advantage in B16-OVA-bearing mice (Figure 6), and in this regard, primes using chimeric aDEC205 or Ad5 were essentially equivalent. This suggests that aDEC205 chimeric antibodies are a feasible alternative to Ad5 in the context of heterologous prime-boost with an oncolytic rhabdovirus. In addition to overcoming pre-existing immunity, which may be a barrier when using certain viral priming vectors, chimeric aDEC205 antibodies can provide additional practical advantages, including, but not limited to, ease of manufacturing, storage, and the possibility of repeat dosing. This last point is a notable limitation for viral vectors encoding antigens, which generally induce an antiviral immune response after the first dose.

Vaccines employing DCs loaded *ex vivo* with tumor lysate or major histocompatibility complex class I (MHC-I) peptides for re-administration to patients have been studied for decades and have been shown to generate robust memory CD8^+^ T cell responses.^42^ Following research in the 1990s on antigen-loaded DC vaccines, many clinical trials carried out to this end have been unable to achieve significant clinical responses.^43^^,^^44^ Objective response rates for a range of DC vaccines loaded with antigens, such as tyrosinase, gp100, MART-1, and MAGE-A3, and autologous peptides in melanoma patients did not exceed 5%–10%.^45^ With the consideration of limitations and logistical challenges in producing DC vaccines, DC targeting using chimeric antibodies, like aDEC205, may be more feasible for treating a diverse population of patients.^46^ However, as observed in this study (Figure 6E), chimeric aDEC205 antibodies may be insufficient as stand-alone anti-cancer vaccines.

One key feature of chimeric aDEC205 antibodies is that they deliver a specific antigen directly to DCs, which in turn, present antigen and activate CD4^+^ T cells, as well as cross present antigen to CD8^+^ T cells. However, this approach is also not without limitations. For example, there can be antibody/protein engineering challenges restricting the choice of antigen and how many antigens can be fused to a given aDEC205 antibody. This can be somewhat addressed by using more restricted epitopes in tandem or using multiple different chimeric antibodies.

Another consideration for use of chimeric aDEC205 antibodies is the requirement for an adjuvant.^47^ In our study, we found that aDEC205-OVA, administered with poly(I:C) and anti-CD40 adjuvants, was effective in generating anti-OVA responses in mice; however, whereas anti-CD40 antibodies (that target the costimulatory receptor CD40 on DCs to induce their maturation) are highly effective in mice, they have displayed severe toxicity in human cancer immunotherapy trials.^48^^,^^49^ Although this regimen was selected for modeling purposes in mice, we expect adjuvants that are amenable to human use and that have been used in clinical trials (e.g., poly(I:C) stabilized with polylysine and carboxymethylcellulose [poly ICLC] Hiltonol) to be similarly effective in combination with aDEC205.^50^ Indeed, many human-compatible adjuvants are known and available and routinely used in the context of cancer vaccines. These include, but are not limited to, alum, poly(I:C), CpG, lipopolysaccharide (LPS), T helper (Th)1-specific cytokines, and growth factors, like Flt3L, important for the development of classical DCs.^40^^,^^51^ These adjuvants, cytokines, and growth factors may be further combined. For example, CDX-301, a soluble recombinant human (rhu)Flt3L, has been used in combination with poly ICLC in the context of a phase II human trial, testing an aDEC205-NY-ESO-1 melanoma vaccination strategy.^52^

Altogether, our study indicates that a vaccine consisting of an aDEC205-OVA prime, followed by a rhabdovirus boost, is a promising alternative to the current heterologous prime-boost that employs Ad5-OVA as a priming agent. To our knowledge, this study is the first of its kind to showcase a combination of the well-studied aDEC205 antibody in combination with an OV. Additional studies in other tumor models and antigenic targets will be necessary to assess the applicability of this novel approach to a broad range of disease models.

## Materials and Methods

### Cell Lines

HEK 293T cells, kindly donated by the Oncolytic Virus Manufacturing Facility (OVMF; Ottawa, Canada) for antibody production and purification, were cultured in HyQ high-glucose Dulbecco’s modified Eagle’s medium (HyClone), supplemented with 10% ultra-low IgG fetal bovine serum (FBS; Gibco), 5% penicillin/streptomycin (pen-strep; Gibco), and 5% L-glutamine (Gibco). B16-F10-OVA cells, kindly gifted by Dr. Yonghong Wan (McMaster University), were cultured in Roswell Park Memorial Institute (RPMI; HyClone), supplemented with 10% FBS, pen-strep, 1 M HEPES buffer, and 50 μg/mL geneticin sulfate (G148 sulfate) (Gibco). All cell lines were incubated at 37°C in a 5% CO_2_ humidified incubator. All cells were tested by PCR and Hoechst staining to ensure that they are free of mycoplasma contamination.

### Mice

6- to 8-week-old female C57BL/6J mice were obtained from Charles River Laboratories. All animals were handled in strict accordance with good animal practice and approved by the appropriate committee in collaboration with the Office of Animal Ethics and Compliance.

### Antibody Production and Purification

The pcDNA plasmids expressing the heavy-chain aDEC205, aDEC205-OVA, and aDEC205-empty and the light-chain DEC205-kappa sequences were generated by Dr. Silvia Boscardin (University of São Paulo). The plasmid DNA was individually transformed in competent DH5-α, and DNA was purified using the QIAGEN Plasmid Maxi Kit (catalog [Cat.] 12165). Transfection of 90% confluent HEK293T cells in 150 mm tissue-culture dishes, collection of antibody from culture supernatant, and antibody purification were performed as previously described.^53^

### Peptides

Peptides corresponding to the immunodominant epitope of OVA (SIINFEKL) that binds to H-2K^b^ were synthesized by New England Peptide (lot number 3001-1/48-21) and have >95% purity.

### Tissue Processing

SIINFEKL-specific T cell responses were measured in blood, spleen, and lung. Briefly, saphenous bleeds of mice from hindlimb were performed, and blood (70–100 μL) was collected in sterile heparin tubes. Red blood cells were lysed using ammonium-chloride-potassium (ACK) lysis buffer. Spleens were excised from sacrificed mice and filtered through a 100-μm plastic cell strainer (Fisherbrand; 352360, 22-363-549) for cell collection. The cell viability of the resulting white blood cells was determined using Trypan blue staining. Lungs were also excised from sacrificed mice after lung perfusion and dissociated using the Lung Dissociation Kit-Mouse (Miltenyi Biotec; 130-095-927), according to the manufacturer’s instructions. Upon resuspension in R10 buffer (RPMI, 10% FBS), the cells from blood, spleen, and lung were counted, and 1 × 10^6^ cells per condition were stained for flow cytometry.

### Immunoblotting

After aDEC205-OVA antibody quantification by the NanoDrop ND-1000 spectrometer, 1 μg of antibody was run on NuPAGE Novex 4%–12% Bis-Tris precast gels (Thermo Fisher Scientific) under reducing conditions using the XCell SureLock Mini-Cell System (Thermo Fisher Scientific) and transferred to nitrocellulose membranes (Hybond-C; Bio-Rad). Blots were blocked with 2% milk and probed with a goat anti-mouse peroxidase-conjugated antibody (1:2,000) (Jackson ImmunoResearch Laboratories). Bands were visualized using the SuperSignal West Pico Chemiluminescent substrate (Thermo Fisher Scientific).

### ELISA

Murine serum was collected from blood for detection of OVA-specific antibodies. Briefly, blood (500 μL) from immunized mice was collected in sterile, 1.5 mL Eppendorf tubes. Collected blood was centrifuged for 10 min at 2,000 × *g*, and the resulting serum in the supernatant was collected and frozen at −20°C for downstream use. Murine serum samples were evaluated for presence of OVA-specific antibodies by ELISA for all groups. 96-well enzyme immunoassay (EIA)/radioimmunoassay (RIA) microplates (Corning; Cat. CLS3590) were coated with albumin (Sigma-Aldrich; A5503-1G) at a concentration of 2 ng/μL in PBS and incubated overnight at 4°C. Plates were washed twice with PBS-Tween 20 0.02% and blocked with blocking buffer (PBS-Tween 20 0.02%, 5% nonfat milk, and 1% BSA) for 1 h at room temperature (RT). Blocking buffer was removed, and serum dilutions (1:500–1:1,000,000 dilution in PBS-Tween 20 0.02%, 5% nonfat milk, and 0.25% BSA) were added to wells and incubated for 2 h at RT. Plates were washed three times with PBS-Tween 20 0.02%, and horseradish peroxidase (HRP)-AffiniPure goat anti-mouse IgG (Jackson ImmunoResearch), diluted 1:4,000, was added to wells and incubated for 1 h at RT. Plates were washed six times with PBS-Tween 20 0.02%, developed with substrate solution (R&D Systems; Cat. DY99), and incubated for 20 min in the dark (RT); development was stopped by addition of 2 N sulfuric acid, and absorbance was read at 510 nm on a Multiskan Ascent plate reader (Thermo LabSystems).

### Neutralization Assay

A neutralization assay was performed to quantify the amount neutralizing antibodies against WTAd5, present in serum samples of preimmunized murine, and is based on the ability of serum antibodies to block adenovirus infection of A549 cells. Adenovirus used carries the firefly luciferase (Fluc) reporter gene, E1 deletion, and cytomegalovirus (CMV) promoter. 2-fold serum dilutions (1:100; 1:200; 1:400; 1:800; 1:1,600; 1:3,200; 1:6,400; 1:12,800; 1:25,600; 1:51,200; 1:102,400) were tested. In 96-well flat-bottom plates, the Ad-Fluc virus (MOI 100) was combined with different serum dilutions and incubated for 1 h at 37°C. Contents of this plate were transferred to a 96-well flat-bottom plate, previously seeded with 2 × 10^5^ A549 cells per well, washed 3× with PBS, and incubated for 48 h at 37°C. To read plate, luciferin was added at a final concentration of 2 mg/mL luciferin per well and imaged/read by the Biotek Synergy Mx Microplate Reader. The antibody neutralizing unit (NU) was defined as the minimum serum dilution required to achieve at least an 80% reduction in luciferase activity, which was assumed to correlate directly to an inhibition of vector infection.

### Mouse Tumor Model and Injections

B16-OVA lung tumors were established in 8-week-old female C57BL/6 mice by i.v. injection of 3 × 10^5^ cells in 100 μL PBS. For adenovirus injections, mice were anesthetized with 5% isoflurane. WTAd5 (10^10^ PFUs) and rAd5-SIINFEKL (10^8^ PFUs) were administered i.m. in 50 μL PBS. For aDEC205 injections, a solution containing 10 μg of aDEC205, 50 μg poly(I:C), and 50 μg anti-CD40 ligand (CD40L) in 150 μL of PBS was administered either i.v. or i.p. Oncolytic rhabdoviruses (MG1-OVA and VSVΔ51-OVA) were administered i.v. in 100 μL of PBS.

### Detection of Antigen-Specific T Cell Responses

OVA-specific T cell responses were measured 7 days and 14 days postboost in blood, spleen, and lung. Splenocytes and lung-resident lymphocytes were isolated and stained for the presence of SIINFEKL-specific T cells using a H-2K^b^-SIINFEKL pentamer. For SIINFEKL-specific CD8^+^ T cell *in vitro* restimulation, 1 × 10^6^ splenocytes and lung-resident lymphocytes were incubated in RPMI medium, supplemented with 10% FBS and 5% pen-strep containing 5 μM of SIINFEKL peptide and brefeldin A (Golgi plug) for 4 h. ICS was performed as described below.

### Virus Preparation

The adenoviruses were made using standard techniques.^54^ The Indiana serotype of VSV (VSVΔ51 or VSVΔ51-OVA) and the Brazilian MG1 (or MG1-OVA) were used throughout this study and were propagated in Vero cells. VSVΔ51-expressing and MG1-expressing OVA are recombinant derivatives of VSVΔ51 and MG1, described previously.^55^ All viruses were propagated on Vero cells and purified on 5%–50% OptiPrep (Sigma) gradient, and all virus titers were quantified by the standard plaque assay on Vero cells, as previously described.^56^

### Antibody Binding Assay

A flow cytometry-based binding assay was performed for evaluation of aDEC205-OVA and aDEC205-empty binding specificity to the target DEC205 receptor on DCs. Bulk splenocytes were isolated from spleens of naive C57BL/6J mice. Red blood cells were lysed, and 5 × 10^6^ bulk splenocytes were incubated with graded concentrations of antibody (0.1 μg/μL, 1 μg/μL, and 10 μg/μL) in a 96-well plate for 45 min (4°C). After incubation, cells were stained for flow cytometry.

### Flow Cytometry

After processing the tissues as described above, cells were then stained with the FVS780 viability dye (BD Biosciences, San Jose, CA) PBS for 15 min at RT. Following washes, cells were incubated with anti-CD16/32 in 0.5% BSA/PBS at 4°C to block nonspecific antibody interaction with Fc receptors. Subsequently, the following protocols were used for staining.

#### Staining for Antibody Binding Assay

Anti-CD11c-phycoerythrin (PE)-Cy7, anti-MHC-I-PE, anti-CD8-PE-CF594, anti-IgG-allophycocyanin (APC), anti-CD3-fluorescein isothiocyanate (FITC), and anti-CD19-FITC antibodies were added to cells and incubated for 30 min (4°C).

#### Staining for ICS

First, 1 × 10^6^ cells were incubated with antibodies targeting T cell surface markers CD3-AF700 and CD8-PE-CF594 for 30 min (4°C). Cells were washed twice with fluorescence-activated cell sorting (FACS) buffer. Next, the mouse Cytofix/Cytoperm Plus (BD Bioscience) was used for permeabilization and ICS. Cells were incubated with Cytofix for 20 min to permeabilize cells for ICS (4°C). Cells were washed twice with PermWash and incubated with anti-IFN-γ-BV650 and anti-TNF-α-AF647 diluted in PermWash for 30 min (4°C).

#### Staining for OVA-Specific T Cells/Pentamer Staining

Cells were washed with FACS buffer. In a 96-well plate, 3 μL of H-2K^b^-SIINFEKL pentamer-APC (Proimmune) in 50 μL of FACS buffer was added per well and incubated for 10 min (RT) in the dark. Cells were washed twice with FACS buffer and stained with fixable viability stain for 30 min (4°C). Subsequently, the cells were washed with FACS buffer and incubated with anti-CD16/32 in FACS buffer for 5 min (4°C). Next, cells were stained with anti-CD8-PE-CF594 and anti-CD3-AF700 for 30 min (4°C)

After staining, cells were washed with FACS buffer and fixed in 1% paraformaldehyde. Cells were acquired on Becton Dickinson (BD) flow cytometry (Fortessa), and analyses were performed using FlowJo software version (v.)9.

### VSV-OVA Cloning and Rescue

Phagemid cloning vector, also known as BlueScribe SK (pBSSK)-VSVΔ51, plasmid-containing viral genome, was used to construct VSVΔ51-OVA. In brief, the OVA gene was PCR amplified from pcDNA expressing aDEC205-OVA using the following primers: forward: 5′-AATTCTCGAGATGGGCTCCATCG-3′ and reverse: 5′-CATCGCTAGCTCACTACAGATCCTC-3′. PCR amplicon was digested by XhoI and NheI and cloned into the multiple cloning site (MCS) of pBSSK-VSVd51 between G and L open reading frames (ORFs). Positive clones were screened by restriction digestion mapping and verified by sequencing.

### Statistics

Statistical significance was calculated using Student’s t test or one-way or two-way ANOVA test, using Tukey’s multiple comparison test, as indicated in the figure legends. The log rank (Mantel-Cox) test was used to determine significant differences in plots for survival studies. Error bars represent standard error of the mean. Significance is based on a p value <0.05. Statistical analyses were performed using GraphPad Prism 6.0 and Excel.

## Author Contributions

F.T. conceived the project. F.T. and J.-S.D. designed the study. All authors participated in the acquisition, analysis, and/or interpretation of data and have read and approved the final manuscript. A.J., H.K.B., and J.-S.D. drafted the manuscript with editorial contributions from F.T. S.B. provided plasmids and protocols. C.T.d.S. and A.C. performed all animal work. M.H. and K.S. constructed and rescued viral vectors. R.J. P. provided adenovirus. J.-S.D. supervised the study.

## Conflicts of Interest

The authors declare no competing interests.

## Supplemental Information

Document S1. Figures S1–S6

Document S2. Article plus Supplemental Information
