# Supplementary material for: Oncolytic Rhabdovirus Vaccine Boosts Chimeric Anti-DEC205 Priming for Effective Cancer Immunotherapy
Source: Mol Ther Oncolytics. 2020 Oct 14;19:240–52. doi: 10.1016/j.omto.2020.10.007 (PMC7658579; doi:10.1016/j.omto.2020.10.007)
Supplement: Document S1. Figures S1–S6 [file mmc1.pdf]

OMTO, Volume *19*

Supplemental Information

Oncolytic Rhabdovirus Vaccine Boosts Chimeric Anti-DEC205 Priming for Effective Cancer Immunotherapy

Fanny Tzelepis, Harsimrat Kaur Birdi, Anna Jirovec, Silvia Boscardin, Christiano Tanese de Souza, Mohsen Hooshyar, Andrew Chen, Keara Sutherland, Robin J. Parks, Joel Werier, and Jean-Simon Diallo

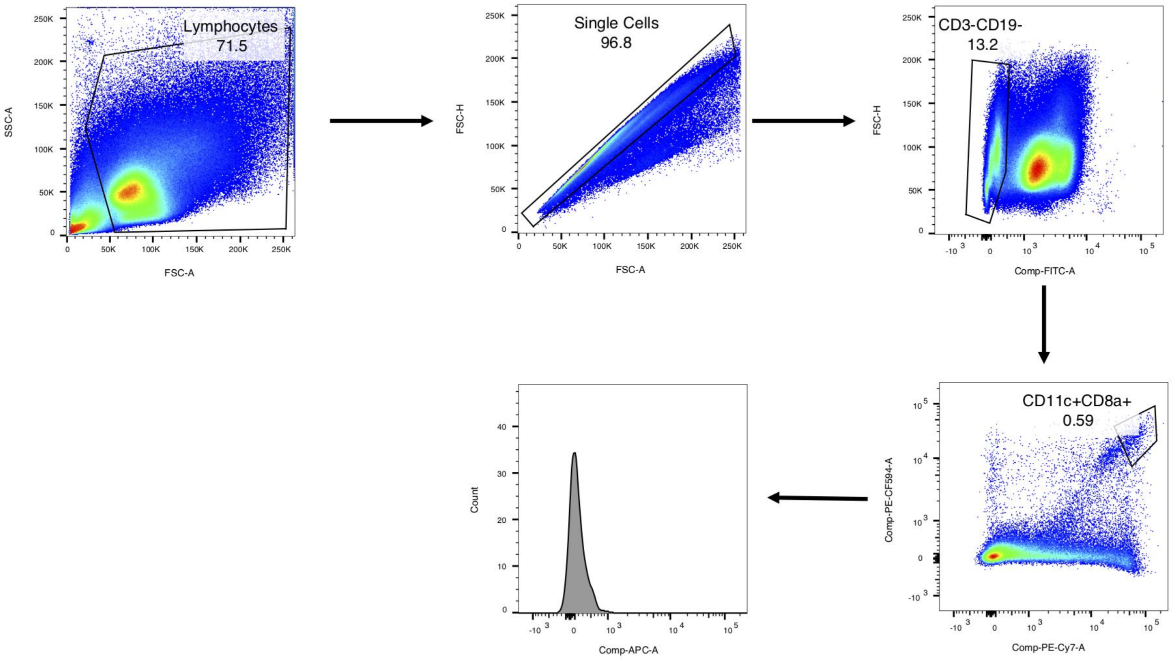

Figure S1. Gating strategy of binding assay to determine the binding efficacy of aDEC205-OVA to CD11c$^+$CD8$^+$ DCs.

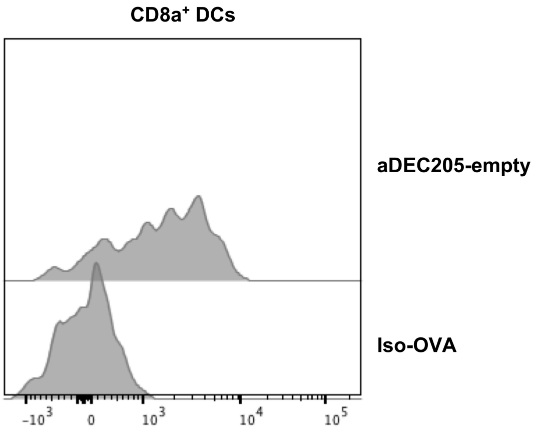

Figure S2. A binding assay was performed to verify effective binding of aDEC205-empty to the DEC205 receptor on CD11c$^+$CD8$^+$ dendritic cells (DCs) isolated from murine splenocytes. aDEC205-empty is probed with an anti-IgG1-APC antibody and detected by flow cytometry. The histogram overlay depicts high binding of aDEC205-empty to CD11c$^+$CD8$^+$ DCs at a concentration of 10μg/mL compared to isotype control.

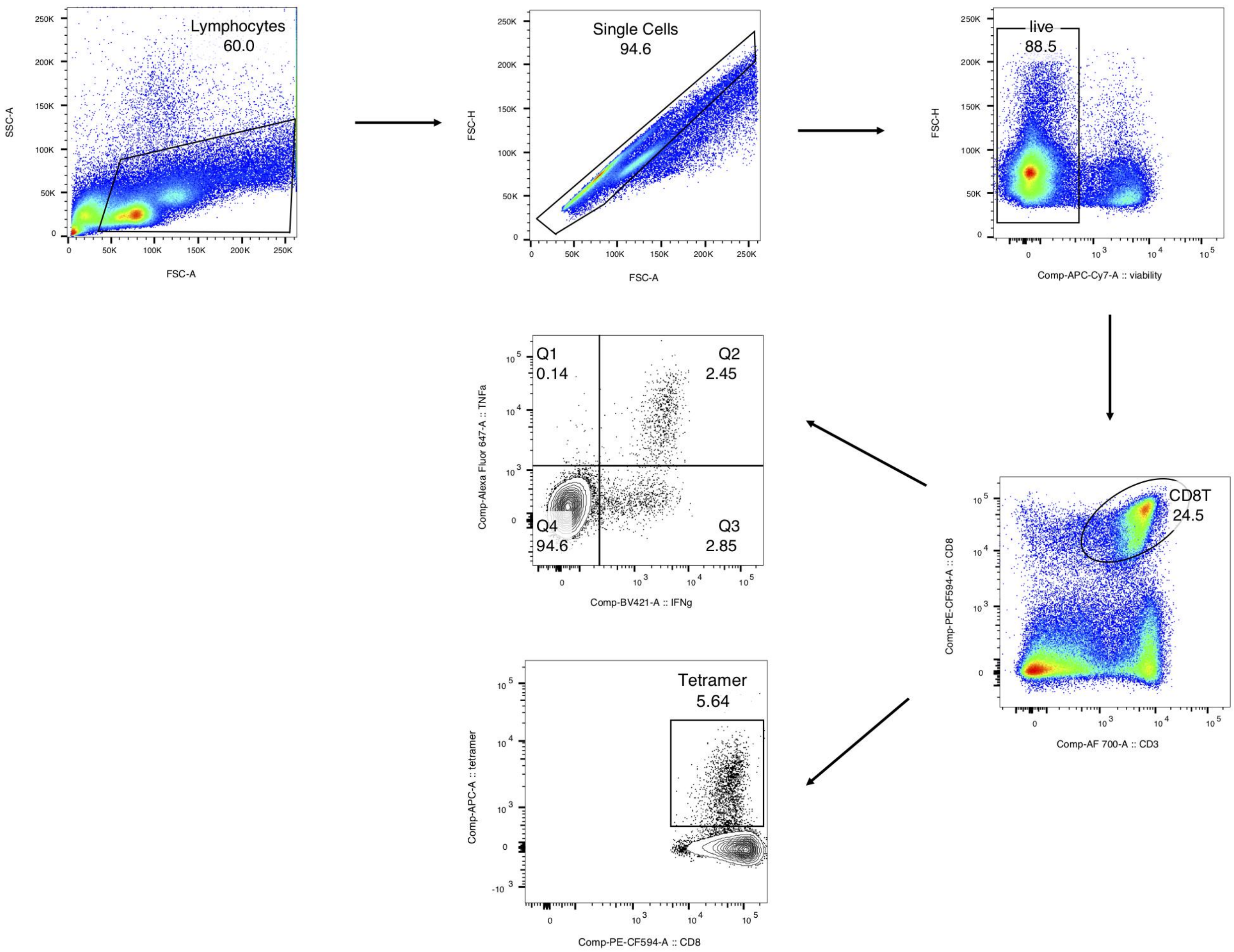

Figure S3. Gating strategy to determine percentage of CD8^{+}CD3^{+} T cells producing IFNγ and TNFα and positive H2-K^{b}-SIINFEKL pentamer staining.

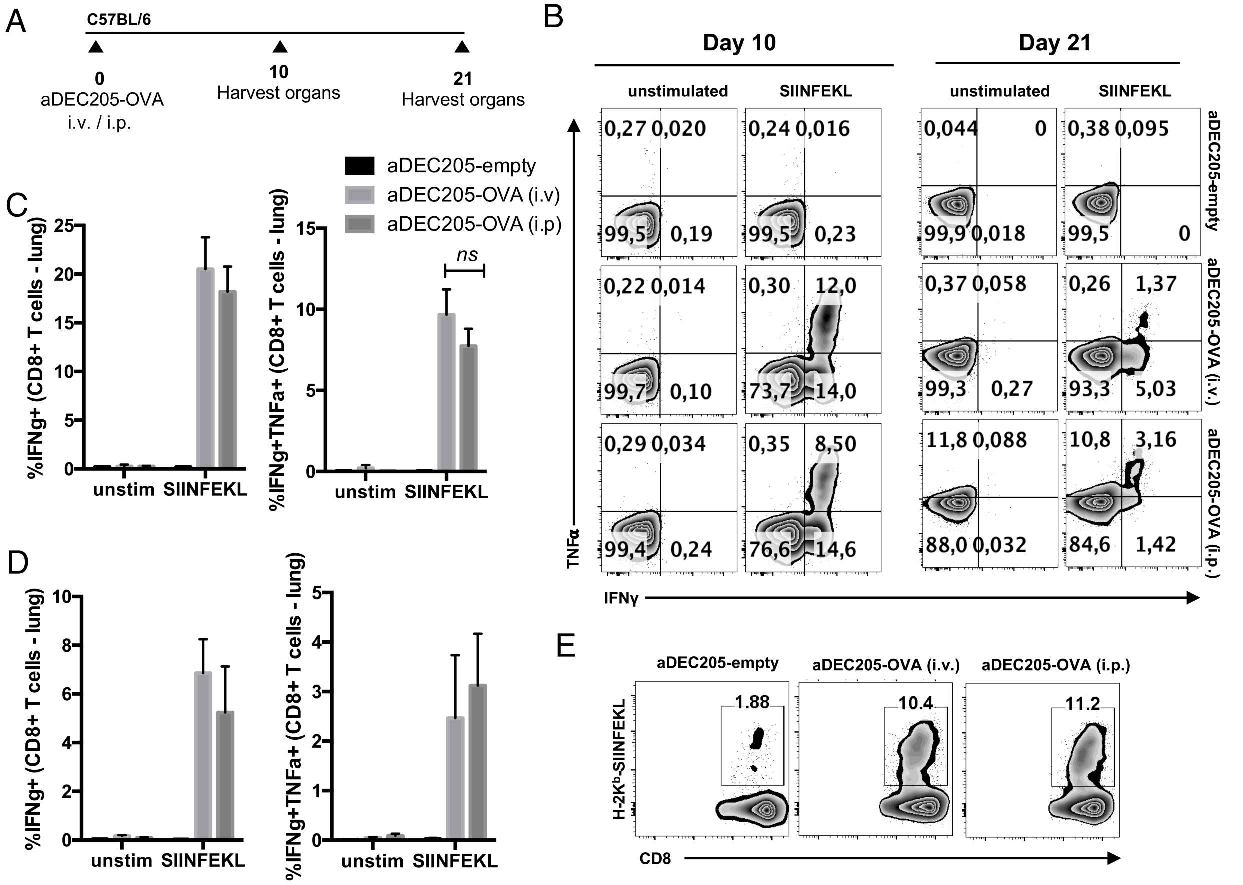

Figure S4. aDEC205-OVA administered i.v. or i.p. elicits OVA specific T cells in the lungs of immunized mice. A. Naïve C57BL/6 mice were primed with 10μg of aDEC205-OVA or aDEC205-empty + 50μg poly:IC + 50μg anti-CD40 i.v. or i.p. The percentage of SIINFEKL-specific T cells producing IFNγ and TNFα in the lungs (**B, C** (D10), **D** (D21)) was evaluated by flow cytometry. Quantification of SIINFEKL specific T cells by pentamer staining (H-$2K^b$-SIINFEKL) was also assessed in the **E.** lungs by flow cytometry at day 21 post injection. *P*-value considered nonsignificant (NS) when >0.05 (two-way ANOVA).

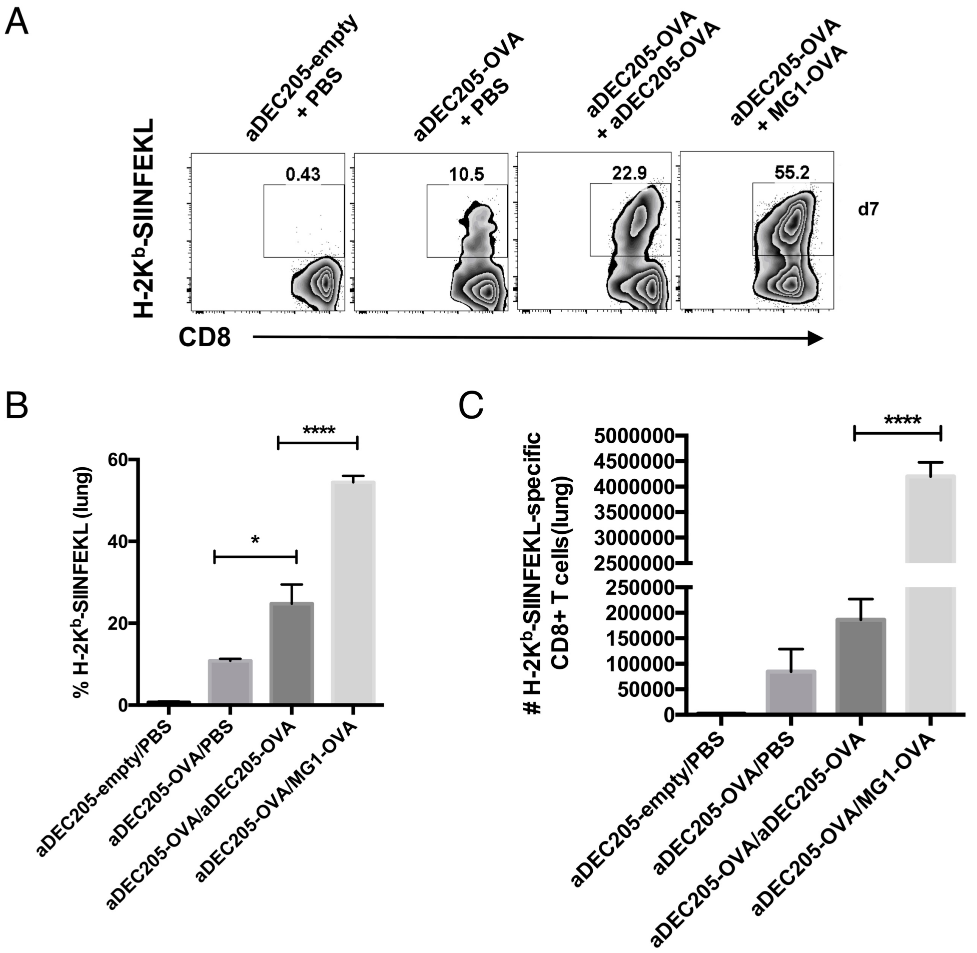

Figure S5. Induction of potent cellular OVA-specific CD8+ T cells in the lungs of mice immunized with aDEC205-OVA prime and MG1-OVA boost. C57BL/6 mice were immunized i.v with 10μg of aDEC205-OVA or aDEC205-empty+50μg poly:IC+50μg anti-CD40 at day 0 (D0). Fourteen days later, mice were immunized with a boosting dose of either, PBS, 10μg aDEC205-OVA i.v +50μg poly:IC+ 50μg anti-CD40 or 10^8 pfu of MG1-OVA. Lungs were harvested 7 days following boost to evaluate cellular immune response to prime-boost regimens. **A,B**. The percentage and **C.** total number of SIINFEKL-specific $CD8^+$ T cells was determined by H2-K^b-SIINFEKL pentamer staining. *, $p<0.05$; **** $p<0.0001$ (one-way ANOVA).

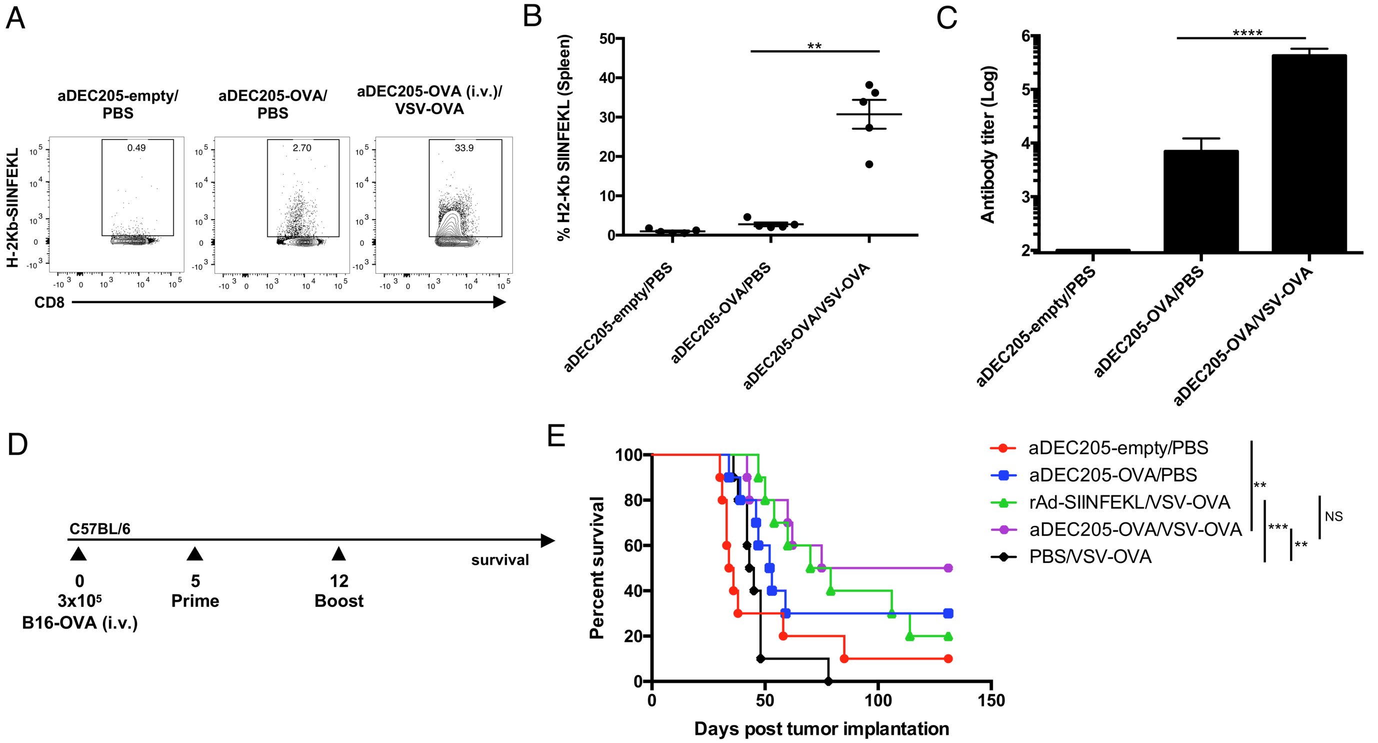

Figure S6. Evaluation of the boosting capacity of VSV-OVA and survival in tumour bearing mice. **A, B.** C57BL/6 mice were immunized i.v with 10μg of aDEC205-OVA or aDEC205-empty+ 50μg poly:IC+50μg anti-CD40 at D0. At day 14 mice received a boosting dose of 10E8 VSV-OVA or PBS. Spleens were harvested on day 21 and assessed for the percentage of OVA-specific CD8+ T cells determined by H-2K^{b}-SIINFEKL pentamer staining. **C.** The titers of anti-OVA antibodies in the sera of mice were determined by ELISA to evaluate the humoral immune response to aDEC205-OVA prime-boost regimen day 7. **D.** C57BL/6 mice were given i.v. injections of $3x10^{5}$ B16-OVA tumour cells. After 5 days, mice were given i.m. injection of rAd5 or i.v. injections of aDEC205-OVA or aDEC205-empty. 7 days post prime, all mice were injected VSV-OVA i.v.. **E.** Mice were monitored for survival 140 days post B16-OVA implantation. $**p < 0.005$, $****p<0.0005$
